# Hypnotics and mortality in an elderly general population: a 12-year prospective study

**DOI:** 10.1186/1741-7015-11-212

**Published:** 2013-09-26

**Authors:** Isabelle Jaussent, Marie-Laure Ancelin, Claudine Berr, Karine Pérès, Jacqueline Scali, Alain Besset, Karen Ritchie, Yves Dauvilliers

**Affiliations:** 1 Inserm, U1061, Montpellier F-34000, France; 2 Université Montpellier 1, Montpellier F-34000, France; 3 Inserm, Centre Inserm U897, Bordeaux F-33000, France; 4 ISPED, Centre Inserm U897, Université Bordeaux, Bordeaux F-33000, France; 5 Faculty of Medicine, Imperial College, London, UK; 6 CHU Montpellier, Service de Neurologie, Unité des Troubles du Sommeil, Hôpital Gui-de-Chauliac, Montpellier, France; 7 Service de Neurologie, Hôpital Gui-de-Chauliac, 80 avenue Augustin Fliche, Montpellier cedex 5 34295, France

**Keywords:** Cohort studies, Elderly, Hypnotics, Mortality, Sleep disorders

## Abstract

**Background:**

Hypnotics are widely used by the elderly, and their impact on mortality remains controversial. The inconsistent findings could be due to methodological limitations, notably the lack of control for underlying sleep symptoms or illness associated with hypnotic use, for example, insomnia symptoms and excessive daytime sleepiness, depression and anxiety. Our objective was to examine the association between the use of hypnotics and mortality risk in a large cohort of community-dwelling elderly, taking into account a wide range of potential competing risks including sociodemographic characteristics, lifestyle, and chronic disorders as well as underlying psychiatric disorders and sleep complaints.

**Methods:**

Analyses were carried out on 6,696 participants aged 65 years or older randomly recruited from three French cities and free of dementia at baseline. Adjusted Cox proportional hazards models with delayed entry, and age of the participants as the time scale, were used to determine the association between hypnotic use and 12-year survival.

**Results:**

At baseline, 21.7% of the participants regularly used at least one hypnotic. During follow-up, 1,307 persons died, 480 from cancer and 344 from cardiovascular disease. Analyses adjusted for study center, age and gender showed a significantly greater risk of all-cause and cardiovascular-related mortality with hypnotics, particularly benzodiazepines, and this increased with the number of hypnotics used. None of these associations were significant in models adjusting for sociodemographic and lifestyle characteristics, chronic disorders including cardiovascular pathologies, sleep and psychiatric disorders. Results remained unchanged when duration of past hypnotic intake or persistent versus intermittent use during follow-up were taken into account.

**Conclusions:**

When controlling for a large range of potential confounders, the risk of mortality was not significantly associated with hypnotic use regardless of the type and duration. Underlying psychiatric disorders appear to be the principal confounders of the observed association.

## Background

Sleep changes with advancing age; however, the high prevalence of insomnia in the older adult population is often due to associated age-related medical and psychosocial comorbidities and the frequent use of medications that may impact sleep *per se*[1]. Insomnia symptoms in older adults are frequently associated with daytime fatigue, excessive daytime sleepiness (EDS), and hypnotics use [2-4]. Insomnia and EDS are also frequently comorbid with other pathologies, notably cardiovascular diseases (CVD) [5,6] and psychiatric disorder, for example, anxiety and depression [2,4,7].

Hypnotics are indicated for treating insomnia symptoms, including those associated with anxiety and depression, and may also be used together with antidepressant treatment. The current use of hypnotics in the general population is estimated to range between 3.5% and 11.7%, doubling in elderly populations [8-11]. Hypnotics may produce residual daytime sleepiness and impairment of psychomotor, attention and memory performances the day after bedtime administration, especially with the high dose and long half-life durations [12]. Moreover, the use of hypnotics seems to be associated with excess risk of accidents such as falls and car accidents [12] and may increase mortality risk, especially in elderly people with increased pharmacodynamic alterations.

However, the high rate of insomnia, EDS complaints, and psychiatric disorder in the elderly, their frequent comorbidity, and the potential risk of mortality associated with both sleep disorders [13] and psychiatric disorders [14,15] may override hypnotics as the cause of increased mortality, independently of the underlying burden of illness.

Overall, evidence suggesting an association between hypnotics consumption and mortality in the elderly remains controversial. Four observational studies in young adults [16,17] and elderly people [18,19] found no significant associations between hypnotics and all-cause mortality. Other studies reported a significant association with excessive all-cause deaths in adults [20-22]. Two large studies with very wide age ranges from young adult to older elderly people [23-25] found significant associations in all age groups, including the elderly. Most of the above studies controlled for sociodemographic characteristics, lifestyle, and some chronic disorders but rarely or not at all for the underlying medical conditions associated with hypnotic prescription, that is depression, antidepressant use, anxiety, insomnia, and EDS. Finally, no studies examined the cumulative effect of hypnotics or the impact of their long-term use on mortality risk in an elderly population specifically. Several methodological issues may contribute to the observed inconsistencies, including the design of the study (retrospective or prospective); the duration of follow-up (between 2.5 and 20 years); the heterogeneity in sample size and age range; the type and duration of hypnotic prescription; and the lack of control for psychiatric and sleep disorders (prescription/indication biases).

The aim of the present study is to examine the associations between the use of hypnotics and 12-year mortality risk (all-causes, cancer and CVD) in a large cohort of community-dwelling elderly people, taking into account a wide range of potential competing risks including sociodemographic characteristics, lifestyle, and chronic disorders as well as underlying psychiatric disorders, EDS, and insomnia complaints. The impact of duration and type of hypnotic treatment were also evaluated.

## Methods

### Study population

Participants were recruited as part of the Three-City Study, an ongoing multi-site longitudinal study involving three French cities: Bordeaux, Dijon and Montpellier [26]. Briefly, non-institutionalized participants aged 65 years or over were randomly selected from electoral rolls between 1999 and 2001. The acceptance rate was 37%, yielding a sample of 9,294 individuals.

The study protocol was approved by the ethical committee of the University Hospital of Kremlin-Bicêtre and CPP Sud Méditérannée III, and written informed consent was obtained from each participant. The participants were administered standardized questionnaires and underwent clinical examinations at baseline and after 2, 4, 8, 10 and 12 years.

### Mortality

The exact date of death of the participants was obtained from death registries. The causes of death were collected by the local study centers from medical records and interviews with family physicians, clinicians and other non-medical informants (relatives or caregivers) [27]. A validation committee used all information to classify the cause of death using the tenth revision of International Classification of Diseases (ICD-10) [28] as follows: cancer (ICD-10: C00 to C97 and D37 to D48), coronary heart disease and stroke (ICD-10: I00 to I99 and R960 to R961), respiratory (ICD-10: J00 to J99), and ill-defined causes (ICD-10: R00 to R99).

### Sociodemographic and clinical variables at baseline

The standardized interview included questions on demographic characteristics, level of education, living alone, and on health behaviors (for example, consumption of alcohol and smoking status). Information on the health of the participants was obtained through detailed medical questionnaires. Case-level depressive symptoms were defined as a score above the 16-point cut-off on the Center for Epidemiological Studies-Depression Scale [29]. Anxiety trait symptoms were measured using the Spielberger’s State-Trait Anxiety Inventory [30]. In the absence of a validated cut-off score in elderly populations, the state score was divided into tertiles with the highest tertile (higher level of anxiety) being compared to the two lowest tertiles. Global cognitive function was assessed by the Mini-Mental State Examination [31] and participants scoring less than 26 were classified as cognitively impaired. Confinement was defined as social restriction (confinement to bed, home or outings restricted to the neighborhood) [32]. Body mass index (BMI) was calculated as weight (kg) divided by height squared (m^2^). The presence of hypertension was defined by measured systolic blood pressure ≥160 mmHg or diastolic blood pressure ≥95 mmHg or current antihypertensive treatment. Diabetes was defined as fasting glucose level ≥7.0 mmol/l or treatment for diabetes. Hypercholesterolemia was defined as total cholesterol level ≥6.2 mmol/L or treatment with lipid-lowering agents. Detailed medical questionnaires included past history of respiratory and thyroid disorders, and cardio-cerebrovascular disease (angina pectoris, myocardial infarction, cardiovascular surgery, arteritis, and stroke) established according to standardized questions.

### Sleep complaints at baseline

Sleep complaints were assessed at baseline as part of the clinical interview, followed by the completion of a specific sleep questionnaire [33]. The participants self-rated as ‘never, rarely, frequently, or often’ occurrence of being excessively sleepy during the day (EDS), having difficulties in initiating sleep (DIS), having several awakenings during the night (difficulties in maintaining sleep; DMS), having early morning awakening (EMA) without being able to go back to sleep, and snoring loudly. In this analysis, EDS was defined as reporting frequently or often being excessively sleepy. Insomnia complaints based on DIS, DMS, and EMA were dichotomized as frequently/often versus never/rarely and summed up to obtain a number of insomnia complaints ranging from 0 to 3. The risk of obstructive sleep apnea syndrome (OSAS) was defined clinically as being obese (BMI ≥30 kg/m^2^), with frequent/often EDS, and frequent/often loud snoring.

### Medications and hypnotic use

At baseline and at 2, 4, and 8-year follow-up, an inventory of all prescriptions and over-the-counter drugs used during the preceding month was included in a standardized interview. Medical prescriptions and the medications themselves were checked by the interviewer, thus minimizing exposure misclassification. Current use of antidepressants and hypnotics were coded according to the World Health Organization’s Anatomical Therapeutic Chemical Classification [34]. Hypnotics were classified as; benzodiazepines (BZD), BZD-like compounds (zolpidem, zopiclone), and miscellaneous medications (including barbiturates, antihistamines, and other pharmacological categories such as neuroleptics). At baseline, the participants currently taking hypnotics were also requested to report the duration of hypnotic intake.

### Statistical analyses

Logistic regression models were used to compare the characteristics of participants according to the use of hypnotics at baseline after adjustment for study center, age, and gender. To analyze the associations between hypnotic use and risk of mortality, Cox proportional hazard models with delayed entry and age of the participants as the time scale were used to estimate hazard ratios (HR) and their 95% confidence intervals (CI). This method gives better adjustment for age and is therefore preferable for a sample of elderly individuals over the standard model that uses study time as the time scale, because the covariates are strongly associated with age (for example, chronic diseases) [35,36]. Multivariate models included covariates that were associated with mortality at a conservative level of *P* <0.15. Model 1 was adjusted for study center, age, and gender. Model 2 was further adjusted for education, alcohol intake, smoking status, BMI, confinement, respiratory disorder, cognitive impairment, history of CVD, hypertension, and diabetes. Two other models were adjusted for diseases associated with hypnotic use to take into account possible prescription bias, for example, number of insomnia complaints and EDS (model 3); and anxiety, depressive symptomatology, and antidepressant use (model 4). The multivariate model 5 was adjusted for all possible confounders. All-cause mortality was the principal outcome defined for the analysis. In secondary analyses, cause-specific mortality due to CVD and cancer was analyzed for separate end points. If both CVD and cancer were reported as cause of death, both causes were considered in the analysis. In all final models, significance level was set at *P* <0.05. Analyses were performed using SAS statistical software (version 9.2; SAS Inc, Cary, NC, USA).

## Results

### Study population

**Figure 1:**
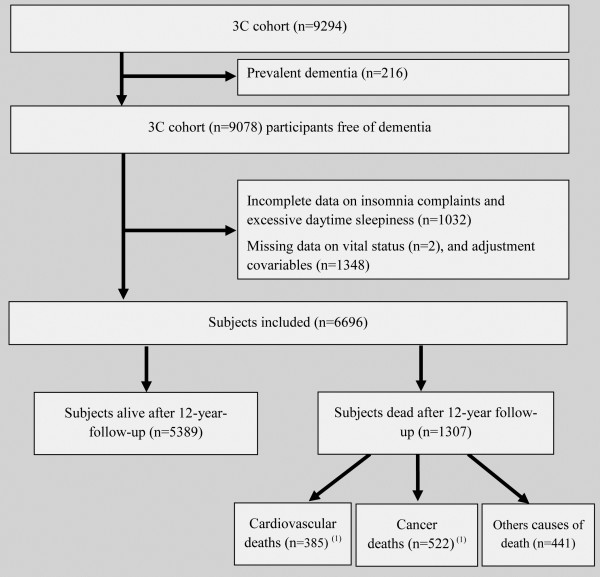
**Flow diagram.**^(1)^ For 41 participants, the cause of death was related to both cardiovascular and cancerb.

As shown in the study diagram (Figure 1), the study sample included 6,696 participants free of dementia (58.7% women) with a median age of 72.8 years (range, 65.0 to 95.0 years). The 2,382 participants free of dementia excluded from the study were significantly more likely to be older, have a lower education level, were more frequently female and living alone, with confinement, hypertension, diabetes, respiratory disease, hypercholesterolemia, depressive and anxiety symptoms, cognitive impairment, past history of cardio-cerebrovascular disease, and taking more hypnotics (*P* <0.05 for all comparisons). They were also more likely to have died during the follow-up period (*P* <0.0001).

At baseline, 21.7% of the participants (n = 1,454) were taking at least one hypnotic, 6.9% (n = 464) had three insomnia complaints, and 3.9% (n = 260) had no insomnia complaints. More than 3% (n = 212) reported taking two or more hypnotics. Regarding the main classes of hypnotics, 16% (n = 1070) took BZD, 4.8% (n = 321) BZD-like compounds, and 3.0% (n = 204) miscellaneous medications (of whom 54.4% took antihistamines, 25.0% non-BZD anxiolytics, 18.6% barbiturates, and 4.4% neuroleptics). With regard to duration, 4.8% (n = 304) had been taking hypnotics for less than 5 years, 3.9% (n = 244) between 5 and 10 years, 2.0% (n = 127) between 10 and 20 years, and 6.0% (n = 378) for more than 20 years.

Baseline sociodemographic and clinical characteristics of the participants according to hypnotic use are described in Table 1. An analysis adjusted for study center, age, and gender showed that participants taking hypnotics had a lower education level; were more likely to be confined to home; had more symptoms of depression, anxiety, and cognitive impairment; more frequently had a past history of chronic disease (CVD, thyroid disease, diabetes, hypercholesterolemia); consumed less caffeine; and reported more insomnia complaints and EDS (*P* <0.05 for all comparisons).

**Table 1:** Sociodemographic and clinical characteristics of participants according to hypnotic use at baseline

		**Hypnotic use**					
		**No N = 5,242**		**Yes N = 1,454**			
**Variable**		**n**	**%**	**n**	**%**	**Odds ratio [95% CI]**^**a**^	***P***
High level of education^b^	No	4,136	78.90	1,245	85.63	1	0.0006
	Yes	1,106	21.10	209	14.37	0.75 [0.63;0.88]	
Living alone	Yes	1,610	30.71	605	41.61	1	0.06
	No	3,632	69.29	849	58.39	0.88 [0.77;1.01]	
Confinement	No	5,025	95.86	1,314	90.37	1	<0.0001
	Yes	217	4.14	140	9.63	1.90 [1.51;2.40]	
Alcohol intake (g/day)	<12	955	18.22	349	24.00	1	0.14
	12 to 36	3,799	72.47	1,012	69.60	0.86 [0.75;1.00]	
	>36	488	9.31	93	6.40	0.92 [0.70;1.22]	
Caffeine intake (mg/day)	≤125	1,310	24.99	423	29.09	1	0.007
	125 to 375	3,103	59.19	836	57.50	0.83 [0.73;0.95]	
	>375	829	15.81	195	13.41	0.76 [0.62;0.92]	
Smoking status	Never	3,033	57.86	969	66.64	1	0.38
	Past	1,901	36.26	402	27.65	0.99 [0.85;1.15]	
	Current	308	5.88	83	5.71	1.19 [0.92;1.55]	
History of cardiovascular disease^c^	No	3,902	74.44	945	64.99	1	<0.0001
	Yes	1,340	25.56	509	35.01	1.59 [1.40;1.81]	
Respiratory disease	No	4,945	94.33	1,364	93.81	1	0.35
	Yes	297	5.67	90	6.19	1.13 [0.88;1.44]	
Thyroid disease	No	4,823	92.01	1,286	88.45	1	0.009
	Yes	419	7.99	168	11.55	1.29 [1.07;1.57]	
Depressive symptomatology	No	4,295	81.93	897	61.69	1	<0.0001
	Yes	947	18.07	557	38.31	2.76 [2.41;3.15]	
Antidepressants intake	No	5,081	96.93	1,196	82.26	1	<0.0001
	Yes	161	3.07	258	17.74	6.32 [5.12;7.81]	
Spielberger trait anxiety	<43	3,688	70.35	715	49.17	1	<0.0001
	≥43	1,554	29.65	739	50.83	2.33 [2.06;2.64]	
Mini Mental State Examination Score	≥ 26	4,559	86.97	1,190	81.84	1	<0.0001
	<26	683	13.03	264	18.16	1.40 [1.20;1.65]	
Body mass index (kg/m^2^)	Normal (<25)	2,477	47.25	747	51.38	1	0.41
	Overweight (25 to 29)	2,106	40.18	515	35.42	0.92 [0.81;1.05]	
	Obese (≥30)	659	12.57	192	13.20	1.00 [0.83;1.20]	
Hypertension	No	2,175	41.49	538	37.00	1	0.08
	Yes	3,067	58.51	916	63.00	1.12 [0.99;1.27]	
Diabetes mellitus	No	4,777	91.13	1,314	90.37	1	0.03
	Yes	465	8.87	140	9.63	1.25 [1.02;1.54]	
Hypercholesterolemia	No	3,349	63.89	859	59.08	1	0.005
	Yes	1,893	36.11	595	40.92	1.19 [1.05;1.34]	
Snoring loudly (n = 5,972)	Never/Rarely	3,021	64.30	863	67.74	1	0.63
	Frequently/Often	1,677	35.70	411	32.26	0.97 [0.84;1.11]	
Difficulties in initiating sleep	Never/Rarely	3,760	71.73	641	44.09	1	<0.0001
	Frequently/Often	1,482	28.27	813	55.91	2.85 [2.51;3.24]	
Difficulties in maintaining sleep	Never/Rarely	2,029	38.71	455	31.29	1	<0.0001
	Frequently/Often	3,213	61.29	999	68.71	1.29 [1.14;1.47]	
Early morning awakening	Never/Rarely	3,565	68.01	740	50.89	1	<0.0001
	Frequently/Often	1,677	31.99	714	49.11	1.85 [1.64;2.09]	
Number of insomnia complaints^d^	0	1,586	30.26	260	17.88	1	<0.0001
	1	1,729	32.98	326	22.42	1.15 [0.96;1.38]	
	2	1,138	21.71	404	27.79	2.02 [1.70;2.41]	
	3	789	15.05	464	31.91	3.07 [2.56;3.68]	
Excessive daytime sleepiness	Never/Rarely	4,384	83.63	1,165	80.12	1	0.005
	Frequently/Often	858	16.37	289	19.88	1.25 [1.07;1.47]	

^a^Adjustment for center study, age and gender; ^b^university level; ^c^history of cardiovascular disease (stroke or coronary heart disease); ^d^number of insomnia complaints: difficulties in initiating sleep + difficulties in maintaining sleep + early morning awakening. CI, confidence interval.

### Association between hypnotic use and 12-year mortality

The median follow-up time for the study was 8.9 years with a range of 0.06 to 11.7 years. During this period, 1,307 (19.5%) deaths were observed. They were particularly related to CVD (26.3%), cancer (36.8%), and co-morbid CVD and cancer (3.1%). A substantial number of deaths were due to ill-defined causes (21.6%) as the result of multiple pathologies associated with frailty, and 10.2% died from respiratory diseases.

Baseline sociodemographic and clinical characteristics in relation to follow-up mortality (all causes) are given in Table 2. Participants who died during follow-up were more frequently confined to home, obese, past or current smoker, consuming less alcohol, had hypertension, diabetes mellitus, a past history of CVD, respiratory disease, poorer cognitive performance, EDS, depressive symptoms, or were taking antidepressants. They also tended to have a lower level of education, and more frequently reported insomnia and anxiety symptoms (*P* <0.15). Subsequent analyses were adjusted for these factors. A significant association was also found for participants at risk of OSAS (n = 133; HR = 1.76; 95% CI = 1.31, 2.36; *P* = 0.0002).

**Table 2:** Baseline predictors of deaths from all causes during follow-up

		**Deaths-all causes**					
		**No N = 5,389**		**Yes N = 1,307**			
**Variable**		**n**	**%**	**n**	**%**	**Hazard ratio [95% CI]**^**a**^	***P***^**a**^
High level of education^b^	No	4,337	80.48	1,044	79.88	1	0.06
	Yes	1,052	19.52	263	20.12	0.87 [0.76;1.00]	
Living alone	Yes	1,782	33.07	433	33.13	1	0.84
	No	3,607	66.93	874	66.87	1.01 [0.89;1.15]	
Confinement	No	5,181	96.14	1,158	88.60	1	0.0001
	Yes	208	3.86	149	11.40	1.78 [1.49;2.13]	
Alcohol intake (g/day)	<12	1,062	19.71	242	18.52	1.23 [1.06;1.42]	0.01
	12 to 36	3,900	72.37	911	69.70	1	
	>36	427	7.92	154	11.78	1.14 [0.96;1.36]	
Caffeine intake (mg/day)	≤125	1,362	25.27	371	28.39	1	0.86
	125 to 375	3,164	58.71	775	59.30	0.97 [0.85;1.09]	
	>375	863	16.01	161	12.32	0.97 [0.81;1.17]	
Smoking status	Never	3,368	62.50	634	48.51	1	<0.0001
	Past	1,740	32.29	563	43.08	1.22 [1.07;1.40]	
	Current	281	5.21	110	8.42	1.73 [1.41;2.14]	
History of cardiovascular disease^c^	No	4,079	75.69	768	58.76	1	0.0001
	Yes	1,310	24.31	539	41.24	1.49 [1.33;1.67]	
Respiratory disease	No	5,121	95.03	1,188	90.90	1	0.0001
	Yes	268	4.97	119	9.10	1.64 [1.36;1.98]	
Thyroid disease	No	4,890	90.74	1,219	93.27	1	0.19
	Yes	499	9.26	88	6.73	1.16 [0.93;1.45]	
Depressive symptomatology	No	4,209	78.10	983	75.21	1	0.0005
	Yes	1,180	21.90	324	24.79	1.26 [1.11;1.43]	
Antidepressant use	No	5,072	94.12	1,205	92.20	1	0.0002
	Yes	317	5.88	102	7.80	1.47 [1.20;1.80]	
Spielberger trait anxiety	<43	3,509	65.11	894	68.40	1	0.13
	≥43	1,880	34.89	413	31.60	1.10 [0.97;1.23]	
Mini Mental State Examination Score	≥ 26	4,666	86.58	1,083	82.86	1	0.005
	<26	723	13.42	224	17.14	1.23 [1.07;1.43]	
Body mass index (kg/m^2^)	Normal (<25)	2,611	48.45	613	46.90	1	0.0007
	Overweight (25 to 29)	2,118	39.30	503	38.49	1.01 [0.90;1.14]	
	Obese (≥30)	660	12.25	191	14.61	1.36 [1.15;1.60]	
Hypertension	No	2,313	42.92	400	30.60	1	0.002
	Yes	3,076	57.08	907	69.40	1.21 [1.07;1.36]	
Diabetes mellitus	No	4,965	92.13	1,126	86.15	1	0.0001
	Yes	424	7.87	181	13.85	1.58 [1.35;1.85]	
Hypercholesterolemia	No	3,333	61.85	875	66.95	1	0.99
	Yes	2,056	38.15	432	33.05	1.00 [0.89;1.12]	
Snoring loudly (n = 5,972)	Never/Rarely	3,114	64.93	770	65.48	1	0.90
	Frequently/Often	1,682	35.07	406	34.52	1.01 [0.89;1.14]	
Difficulties in initiating sleep	Never/Rarely	3,506	65.06	895	68.48	1	0.20
	Frequently/Often	1,883	34.94	412	31.52	0.92 [0.81;1.04]	
Difficulties in maintaining sleep	Never/Rarely	2,055	38.13	429	32.82	1	0.26
	Frequently/Often	3,334	61.87	878	67.18	1.07 [0.95;1.20]	
Early morning awakening	Never/Rarely	3,434	63.72	871	66.64	1	0.12
	Frequently/Often	1,955	36.28	436	33.36	0.91 [0.81;1.02]	
Number of insomnia complaints^d^	0	1,526	28.32	320	24.48	1	0.07
	1	1,595	29.60	460	35.20	1.14 [0.99;1.31]	
	2	1,227	22.77	315	24.10	1.08 [0.92;1.26]	
	3	1,041	19.32	212	16.22	0.92 [0.77;1.11]	
Excessive daytime sleepiness	Never/Rarely	4,555	84.52	994	76.05	1	0.003
	Frequently/Often	834	15.48	313	23.95	1.23 [1.07;1.40]	

^a^Adjusted for center study, gender and age; ^b^university level; ^c^history of cardiovascular disease (stroke or coronary heart disease; ^d^number of insomnia complaints: difficulties in initiating sleep + difficulties in maintaining sleep + early morning awakening.

Table 3 shows the associations between hypnotic use at baseline and all-cause mortality over the 12-year follow-up. After adjustment for age, gender, and study center, the risk of all-cause mortality increased significantly with the use of any hypnotic, the number of hypnotics, and alone for BZD (*P* <0.01 for all comparisons, model 1). When potential lifestyle and chronic disorder confounders were entered into the model (model 2), the HR were reduced and failed to be significant except for BZD (*P* = 0.05) and this was unchanged when further adjusting for sleep complaints (model 3). When adjusting for anxiety and depressive symptomatology (model 4), the associations were not significant even for BZD (*P* = 0.22) and this was also the case for the complete multivariate model adjusted for all potential confounders (model 5). BZD-like compounds, and miscellaneous medications intake were not associated with all-cause mortality even in the minimally adjusted model 1. No significant interaction was found for mortality between hypnotic use and EDS, number of insomnia complaints, antidepressant use, chronic diseases, or being at risk for OSAS.

**Table 3:** Risks of death from all causes over 12-year according to hypnotic use

	**All-cause death**													
	**No**		**Yes**		**Model 1**^**a**^		**Model 2**^**b**^		**Model 3**^**c**^		**Model 4**^**d**^		**Model 5**^**e**^	
	**N = 5,389**		**N = 1,307**											
**Variable**	**n**	**%**	**n**	**%**	**HR [95% CI]**	***P***	**HR [95% CI]**	***P***	**HR [95% CI]**	***P***	**HR [95% CI]**	***P***	**HR [95% CI]**	***P***
Hypnotic use														
No	4,261	79.07	981	75.06	1	0.007	1	0.16	1	0.12	1	0.50	1	0.43
Yes	1,128	20.93	326	24.94	1.19 [1.05;1.36]		1.10 [0.96;1.25]		1.12 [0.97;1.29]		1.05 [0.92;1.20]		1.06 [0.92;1.23]	
Number of hypnotics														
0	4,261	79.07	981	75.06	1	0.003	1	0.13	1	0.13	1	0.44	1	0.47
1	970	18.00	272	20.81	1.14 [0.99;1.31]		1.06 [0.93;1.22]		1.09 [0.94;1.26]		1.02 [0.89;1.18]		1.04 [0.89;1.21]	
≥2	158	2.93	54	4.13	1.53 [1.16;2.01]		1.32 [1.00;1.74]		1.33 [0.98;1.81]		1.20 [0.90;1.60]		1.21 [0.88;1.65]	
BZD														
No	4,557	84.56	1069	81.79	1	0.003	1	0.05	1	0.05	1	0.22	1	0.21
Yes	832	15.44	238	18.21	1.24 [1.08;1.44]		1.15 [1.00;1.33]		1.17 [1.00;1.37]		1.10 [0.95;1.28]		1.11 [0.94;1.30]	
BZD-like compounds														
No	5,135	95.29	1240	94.87	1	0.93	1	0.56	1	0.76	1	0.40	1	0.55
Yes	254	4.71	67	5.13	1.01 [0.79;1.29]		0.93 [0.72;1.19]		0.96 [0.74;1.25]		0.90 [0.70;1.15]		0.92 [0.71;1.20]	
Miscellaneous medications														
No	5,241	97.25	1251	95.72	1	0.15	1	0.30	1	0.32	1	0.49	1	0.49
Yes	148	2.75	56	4.28	1.22 [0.93;1.60]		1.15 [0.88;1.51]		1.16 [0.87;1.55]		1.10 [0.84;1.45]		1.11 [0.83;1.48]	

^a^Adjusted for age, study center, and gender; ^b^adjusted for age, study center, gender, level of education, confinement, alcohol intake, smoking status, history of cardio and cerebrovascular disease, respiratory disease, Mini Mental State Examination score, body mass index, hypertension, and diabetes mellitus; ^c^adjusted for all the covariates in model 2, plus excessive daytime sleepiness and number of insomnia complaints; ^d^adjusted for all the covariates in model 2, plus depressive symptoms, antidepressant use and Spielberger trait anxiety score; ^e^adjusted for all the covariates in model 3 plus depressive symptoms, antidepressants use and Spielberger trait anxiety score. BZD, benzodiazepines; CI, confidence interval; HR, hazard ratios.

The relationship between hypnotic intake and the risk of mortality remained unchanged after exclusion of the participants who died during the first two years of follow-up (n = 134), the follow-up rate at two years being 88%. With regard to specific causes of death, the use of hypnotics and BZD as well as number of hypnotics were associated with a significantly increased risk of CVD-related death in model 1, but not in the complete multivariate model adjusted for all potential confounders (Table 4). There was no significant association between hypnotics and cancer-related death regardless of covariates, even in the minimally-adjusted model 1 (*P* = 0.38).

**Table 4:** Risks of cardiovascular disease and cancer as causes of death over 12 years according hypnotic use

	**Cardiovascular disease deaths**								**Cancer deaths**							
	**No n = 6,311**		**Yes N = 385**		**Model 1**^**a**^		**Model 2**^**b**^		**No n = 6,174**		**Yes N = 522**		**Model 1**^**a**^		**Model 2**^**b**^	
**Variable**	**n**	**%**	**n**	**%**	**HR [95% CI]**	***P***	**HR [95% CI]**	***P***	**n**	**%**	**n**	**%**	**HR [95% CI]**	***P***	**HR [95% CI]**	***P***
Hypnotic use																
No	4,962	78.62	280	72.73	1	0.02	1	0.56	4,818	78.04	424	81.23	1	0.38	1	0.73
Yes	1,349	21.38	105	27.27	1.32 [1.04;1.66]		0.92 [0.71;1.20]		1,356	21.96	98	18.77	0.90 [0.72;1.13]		0.96 [0.74;1.23]	
Number of hypnotics																
0	4,962	78.62	280	72.73	1	0.03	1	0.78	4,818	78.04	424	81.23	1	0.67	1	0.94
1	1,154	18.29	88	22.86	1.26 [0.99;1.61]		0.94 [0.72;1.23]		1,157	18.74	85	16.28	0.91 [0.72;1.15]		0.96 [0.74;1.25]	
≥2	195	3.09	17	4.42	1.69 [1.03;2.76]		0.83 [0.45;1.52]		199	3.22	13	2.49	0.89 [0.51;1.54]		0.93 [0.50;1.72]	
BZD																
No	5,321	84.31	305	79.22	1	0.004	1	0.60	5,170	83.74	456	87.36	1	0.27	1	0.42
Yes	990	15.69	80	20.78	1.45 [1.13;1.87]		1.08 [0.81;1.43]		1,004	16.26	66	12.64	0.86 [0.66;1.12]		0.89 [0.66;1.19]	
BZD-like compounds																
No	6,008	95.20	367	95.32	1	0.65	1	0.12	5,879	95.22	496	95.02	1	0.78	1	0.44
Yes	303	4.80	18	4.68	0.90 [0.56;1.44]		0.67 [0.40;1.11]		295	4.78	26	4.98	1.06 [0.71;1.57]		1.18 [0.77;1.82]	
Miscellaneous medications																
No	6,123	97.02	369	95.84	1	0.64	1	0.43	5,986	96.95	506	96.93	1	0.88	1	0.95
Yes	188	2.98	16	4.16	1.13 [0.68;1.87]		0.80 [0.45;1.41]		188	3.05	16	3.07	0.96 [0.58;1.59]		1.02 [0.59;1.74]	

^a^Adjusted for age, study center, and gender; ^b^adjusted for all covariates in model 1 plus high level of education, confinement, alcohol intake, smoking status, history of cardio-cerebrovascular disease, respiratory disease, Mini Mental State Examination score, body mass index, hypertension and diabetes mellitus, depressive symptoms, antidepressants use, Spielberger trait anxiety score, excessive daytime sleepiness, and number of insomnia complaints. BZD, benzodiazepines; CI, confidence interval; HR, hazard ratios.

### Duration of hypnotic use and mortality

Sensitivity analyses were performed to examine the relationship between persistent use of hypnotics during the initial 4 years and all-cause mortality. A total of 3,496 participants (65.9%) did not report hypnotic use at baseline or at follow-up examination, 773 (14.5%) reported use both at baseline and at the first two follow-ups (persistent users) and 1,040 (19.6%) were taking hypnotics at one of two time points (intermittent users). The risk of mortality for the next 8 years was not significantly associated with the persistent use of hypnotics (when compared with non-users, HR = 1.03, 95% CI = 0.84, 1.28 for intermittent users; HR = 1.11, 95% CI = 0.88, 1.39 for persistent users; multivariate model 5). Similar results were obtained when the analyses focused on persistent BZD users in comparison to non persistent BZD users or non BZD users.

We also examined the impact of past hypnotic intake duration and compared participants who were not taking sleep medication at baseline with those having previously reported taking sleep medications for less than 5 years, between 5 and 10 years, between 10 and 20 years, and for more than 20 years. No significant association was observed between duration of hypnotic intake and all-cause mortality, the global *P*-value ranging from 0.18 (model 1) to 0.76 (model 5) (data not shown).

## Discussion

This study examined associations between hypnotic intake and risk of excess mortality (all-causes and specific causes) over a 12-year period in a large elderly cohort, taking into account a wide range of potential confounding factors. As in several previous studies we observed significant associations between hypnotic use, notably BZDs, and mortality; however, these associations became non-significant after adjustment for all potential confounding factors, notably psychiatric disorder. These findings persisted even after taking into account up to 20 years duration of past hypnotic intake or persistent versus intermittent use.

Previous studies have been inconsistent, with some studies observing significant relationships between hypnotic prescriptions and mortality [16,20-25,37] and others not [16-19]. Our findings suggest that these differences are probably largely due to failure to take into account confounding associations, notably common affective symptoms and sleep complaints, although other factors such as study design, participant age, and class of hypnotics probably also influence study outcome.

Insomnia symptoms often lead to the use of hypnotics, a condition frequently associated with EDS, anxiety, and mood disorders. Depression and anxiety are also risk factors for mortality [14,15]. Depressive symptomatology and insomnia are both common in the elderly and in France there are no official guidelines for management, so antidepressants are often used to treat sleep disorder and hypnotics to treat depression, especially where sleep disturbance is one of the presenting symptoms [2]. EDS is also of multifactorial origin, and commonly associated with depression [7], cognitive decline [38], physical illness (particularly CVD), and mortality in older adults [5,6,13]. Thus, all these conditions may increase the risk of mortality in elderly patients through pathways independently of hypnotics. However, few previous studies have controlled for psychological status [17,18,22,23] and in studies where depressive symptoms have been considered, antidepressant use has not been necessarily taken into account. This is important because antidepressant use may relieve depressive symptomatology, but the underlying biological risk factors associated with increased mortality may still be operating. No previous studies have controlled for anxiety or simultaneously for insomnia and EDS symptoms as potential independent confounding factors. To our knowledge, our study is the first one controlling for such a large range of potential confounding factors, especially the underlying diseases associated with hypnotic use, such as anxiety and depressive symptomatology and antidepressant use, as well as EDS and insomnia complaints. Our finding that psychiatric disorder could be a principal determinant driving the association between hypnotics and mortality risk explains previous inconsistencies.

Chronic use of hypnotic drugs, particularly BZD, may be associated with the risk of addiction and insomnia-rebound after withdrawal, psychomotor impairment and cognitive problems, OSAS, EDS, and car accidents [12,39,40]. In our sample, only one participant died from a car accident, and this person did not use hypnotics. We did not find any interaction between individuals clinically at risk for OSAS, hypnotics intake, and mortality, suggesting that if hypnotics trigger or aggravate OSAS they do not impact on mortality risk. The use of BZD may also favor falls and hip fractures and thus increase the risk for disability and death especially in the elderly [41,42]. However some studies have suggested that nighttime sleep problems may also be significant risk factors for falls in the elderly, independently of hypnotic use [43-45]. In our study, the associations between hypnotic use and all-causes or CVD-related death became non-significant after adjustment for health behavior and status variables, plus EDS and insomnia complaints.

An increased incidence risk for cancer was also reported in individuals using hypnotics in some studies [21,22,24], even in infrequent hypnotic users [22]. Our study did not report any association between hypnotic use and cancer-related death. Again, differences in adjustment of underlying co-morbid conditions frequently associated with the chronic use of hypnotics appear to explain previous findings.

The present study has some limitations. Unfortunately, data related to hypnotic dose were not available. Bias could have been introduced by the low participation rate at baseline and the non-random exclusion of participants with missing data at baseline – these participants were older, were more commonly hypnotics users, and more often had psychiatric and other chronic disorders that may limit the generalizability of our findings. Although unlikely, the possibility of overadjusment can not be excluded: potential confounding variables should be intermediate variables in the causal pathway between hypnotics intake and mortality. Finally, the absence of significant association between the use of hypnotics and mortality (all-causes, and CVD) after adjustments for covariates should be interpreted with caution regarding the small number of events per predictor variable.

Our prospective study based on a large community sample has several strengths, including the duration of the follow-up and adjustment for a wide range of possible confounding factors including sociodemographic and lifestyle factors, chronic disorders, and sleep complaints as well as depression and anxiety disorders that were found as key confounding factors in this study. Prescriptions and medications themselves were checked by the interviewer and the causes of death were established by an independent committee. Finally, excluding participants who died during the first two years of follow-up did not modify the main results, suggesting a modest confounding effect of severe undiagnosed conditions in relation to hypnotic use and death.

## Conclusions

Our findings suggest that the use of hypnotics is not independently associated with an increased risk of mortality in the elderly, and that previous findings may be largely attributable to failure to take into account confounding variables, notably clinical co-morbidity, which is frequent at higher ages, particularly psychiatric disorders. Use of hypnotics might be a marker underlying more complex health issues.

## Abbreviations

BMI: Body mass index; BZD: Benzodiazepines; CI: Confidence interval; CVD: Cardiovascular diseases; DIS: Difficulties in initiating sleep; DMS: Difficulties in maintaining sleep; EDS: Excessive daytime sleepiness; EMA: Early morning awakening; HR: Hazard ratios; ICD-10: International classification of diseases; OSAS: Obstructive sleep apnea syndrome.

## Competing interests

The authors have nothing to disclose in relation to this paper. IJ, MLA, KP, JS and AB report no disclosures. KR has received honoraria from Novartis and Glaxo Smith-Kline; is on the scientific advisory boards for the Biomedical Research Centre, King’s College London, and the MRC Strategic Steering Committee (Longitudinal Health and Aging Research Unit); and serves on the editorial boards of the International Journal of Geriatric Psychiatry, Dementia, International Psychogeriatrics, Journal of Clinical and Experimental Gerontology, Psychogeriatrics, Neuronale, Neurologie-Psychiatrie-Gériatrie, and Gerontology. CB serves on advisory boards of the British Journal of Nutrition and Revue d’épidemiologie et de santé publique. YD has received speaker’s honoraria and support for travel to meetings from UCB Pharma, JAZZ and Bioprojet. YD participated in advisory boards of UCB Pharma, JAZZ and Bioprojet.

## Authors’ contributions

IJ, MLA, and YD participated in the conception and design of the study. IJ conducted the analyses and wrote the first draft of the manuscript. IJ, MLA, AB, YD participated in the interpretation of the data. IJ, MLA, CB, KP, JS, AB, KR and YD contributed to the writing of the manuscript. MLA, CB, KP, JS, KR and YD participated in the acquisition of the data. All authors approved the final manuscript.

## Pre-publication history

The pre-publication history for this paper can be accessed here:

http://www.biomedcentral.com/1741-7015/11/212/prepub
